# Network Pharmacology and Molecular Docking: Exploring the Mechanism of Peppermint in Mastitis Prevention and Treatment in Dairy Cows

**DOI:** 10.3390/vetsci12020129

**Published:** 2025-02-05

**Authors:** Xinyu Wang, Jiaxin Lai, Fei Xu, Mingchun Liu

**Affiliations:** 1 National Feed Drug Reference Laboratories, Institute of Feed Research, Chinese Academy of Agricultural Sciences, Beijing 100081, China; wangxinyu@stu.syau.edu.cn; 2 College of Animal Science and Medicine, Shenyang Agricultural University, Shenyang 110866, China; laijiaxin@stu.syau.edu.cn

**Keywords:** mint, bovine mastitis, network pharmacology, molecular docking, action mechanism

## Abstract

In order to elucidate the active ingredients, potential targets, and mechanisms of action of peppermint in treating bovine mastitis, this study utilized network pharmacology analysis and molecular docking to conduct an exploratory, prospective investigation. Using the Traditional Chinese Medicine Systems Pharmacology (TCMSP) database, all compounds and targets of peppermint were retrieved. After removing duplicates, a total of 133 compounds and 272 targets were obtained. Targets were then standardized to gene names using the UniProt database to construct a drug–component-target network. A total of 183 disease targets related to bovine mastitis were retrieved from the GeneCards database. We obtained 28 cross targets of peppermint targets and bovine mastitis targets, and constructed a protein–protein interaction (PPI) network using the STRING database. A visual network was built using Cytoscape 3.10.0 software, and seven core targets were analyzed and obtained. GO and KEGG pathway enrichment analysis was performed using the Metascape database. Molecular docking was conducted using AutoDockTools–1.5.6 software on some small–molecule compounds and the seven targets to evaluate the stability of binding between peppermint and core targets. Apigenin, luteolin, and ursolic acid are the three main components in peppermint. Core targets (TNF, IL–6, STAT–3, IL–1β, FGF–2, IFNG, and ESR–1) were selected based on the PPI network. The enrichment analysis suggested that the major signaling pathways in network pharmacology may include AGEs–RAGE, IL–17, NF–κB, TLRs, HIF–1, TGF–β, PI3K–Akt, and MAPK. The molecular docking results showed that one of the main components of mint, ursolic acid, exhibited good binding activity with all core targets of bovine mastitis. Other constituents also produced favorable binding with some core targets. This study elucidates the mechanisms of mint in treating bovine mastitis, providing data to support the potential development of new therapies for bovine mastitis using mint and its constituents.

## Simple Summary

Bovine mastitis is a common condition in dairy production units that has serious productive consequences. However, due to the current limitations in the use of antibiotics, alternatives have been sought to counteract the consequences of this pathology, where mint (*Mentha* spp.) has proven to be a viable option. Therefore, this study is designed to elucidate the active ingredients, targets, and mechanisms of action of peppermint in treating bovine mastitis using network pharmacology and molecular docking methods. The findings indicate that the key targets of mint in treating bovine mastitis include Tumor Necrosis Factor (TNF), Interleukin–6 (IL–6), signal transducer and activator of transcription 3 (STAT–3), Interleukin–1 beta (IL–1β), Recombinant Fibroblast Growth Factor 2 (FGF–2), Interferon gamma (IFNG), and Estrogen Receptor 1 (ESR–1), with the primary signaling pathways being the Advanced Glycation End–products–Receptor for Advanced Glycation End–products pathway (AGEs–RAGE), Interleukin–17 (IL–17), the Nuclear Factor kappa–light–chain–enhancer of activated B cells pathway (NF–κB), the Toll–like receptor (TLR) pathway, the hypoxia–inducible factor–1 (HIF–1) pathway, the Transforming Growth Factor–beta pathway (TGF–β), the Phosphatidylinositol 3–kinase–Protein Kinase B pathway (PI3K–Akt), and the mitogen–activated protein kinase pathway (MAPK). Molecular docking results revealed that ursolic acid from mint has good binding activity with all core targets, and other constituents also form stable complexes with some of the core targets. These results suggest that active compounds such as ursolic acid in mint possess positive anti–inflammatory properties. Therefore, mint is considered to have a promising therapeutic effect on bovine mastitis.

## 1. Introduction

The purpose of dairy farming is to produce milk and dairy products. According to data from the Food and Agriculture Organization (FAO) of the United Nations, approximately 81% of the world’s milk is produced by dairy cows, with the remainder coming from buffaloes (15%), goats, sheep, and camels (a combined 4%) [1]. Mastitis is one of the most common diseases in dairy cows, significantly affecting milk yield and quality. In addition to reduced milk production, mastitis also leads to increased treatment costs, higher culling rates of cows, and a decline in milk quality. Besides its economic impact, milk and dairy products, as essential sources of nutrition for humans, play a vital role in dietary health [2]. The majority of mastitis cases in dairy cows are attributed to infections by specific pathogenic bacteria, including *Streptococcus agalactiae*, *Streptococcus dysgalactiae*, *Streptococcus uberis*, *Staphylococcus aureus*, *Corynebacterium bovis*, *Escherichia coli*, *Klebsiella* sp., *Mycoplasma* sp., and *coagulase–negative Staphylococcus* species [3,4]. Therefore, antibiotics are extensively employed in dairy farming to combat infections. However, the irrational and repeated use of antibiotics leads to antimicrobial resistance (AMR) in bacteria, which increases the difficulty and cost of treatment. Moreover, antibiotic residues may enter the human body through the food chain, exposing consumers to low doses of antibiotics. This can cause allergies, drug resistance in humans, and abnormal immune reactions, thereby affecting the healthy development of public health. These factors render the use of antibiotics in the preventive treatment of bovine mastitis unsustainable in the long term [5,6,7]. In summary, bovine mastitis is a key factor leading to increased farming costs and decreased milk quality, posing a significant threat to both economic and public health. Although the dairy industry is almost universally challenged by mastitis in cows, obtaining high–quality milk in dairy farming remains a critically important goal.

For the above reasons, we believe that the use of alternative antibacterial substances, such as plants or plant extracts, has important practical significance in the prevention and treatment of bovine mastitis [8]. Among them, peppermint, as a perennial plant, is recommended for the treatment of mastitis in cows due to its good biological effects [9]. The constituents of mint are primarily categorized into peppermint essential oil (PEO) and other non–essential components. PEO mainly consists of menthol, menthone, neomenthol, and iso–menthone, while other components include flavonoids, triterpenoids, phenolic acids, and others [10,11]. Numerous studies have demonstrated that PEO possesses anti–inflammatory, antibacterial, antiviral, antioxidant, and immunomodulatory activities [12,13,14,15,16,17,18,19]. Additionally, some flavonoid compounds in mint, such as apigenin, luteolin, naringenin, and acacetin, have broad biological effects, including anti–inflammation, anticancer, and antioxidant properties [20,21,22]. Among the pentacyclic triterpenoids, ursolic acid and oleanolic acid also exhibit anti–inflammatory effects [23,24,25]. Phenolic acid compounds, such as rosmarinic acid and caffeic acid, have bacteriostatic and antioxidant activities [26,27]. The research by Safia Arbab also reflects the excellent inhibitory effect of mint extracts on *Staphylococcus aureus* derived from bovine mammary glands, thus recommending the use of mint extracts in combination with other preservatives for the treatment of bovine mastitis [9].

Network pharmacology enables the transformation from a single drug–single target model to a multi–component, multi–target, and multi–pathway research paradigm, thereby revealing the interactions between drugs, genes, and protein targets. This approach is of significant importance for providing more options for the clinical application and in–depth study of mint [28]. Although existing studies have confirmed its efficacy in bovine mastitis, the specific mechanisms are understudied [9]. Therefore, to elucidate the active ingredients, potential targets, and mechanisms of action of peppermint in treating bovine mastitis, this study conducted exploratory research using network pharmacology analysis and molecular docking.

## 2. Materials and Methods

### 2.1. Screening of Active Ingredients and Targets of Peppermint

**Figure 1:**
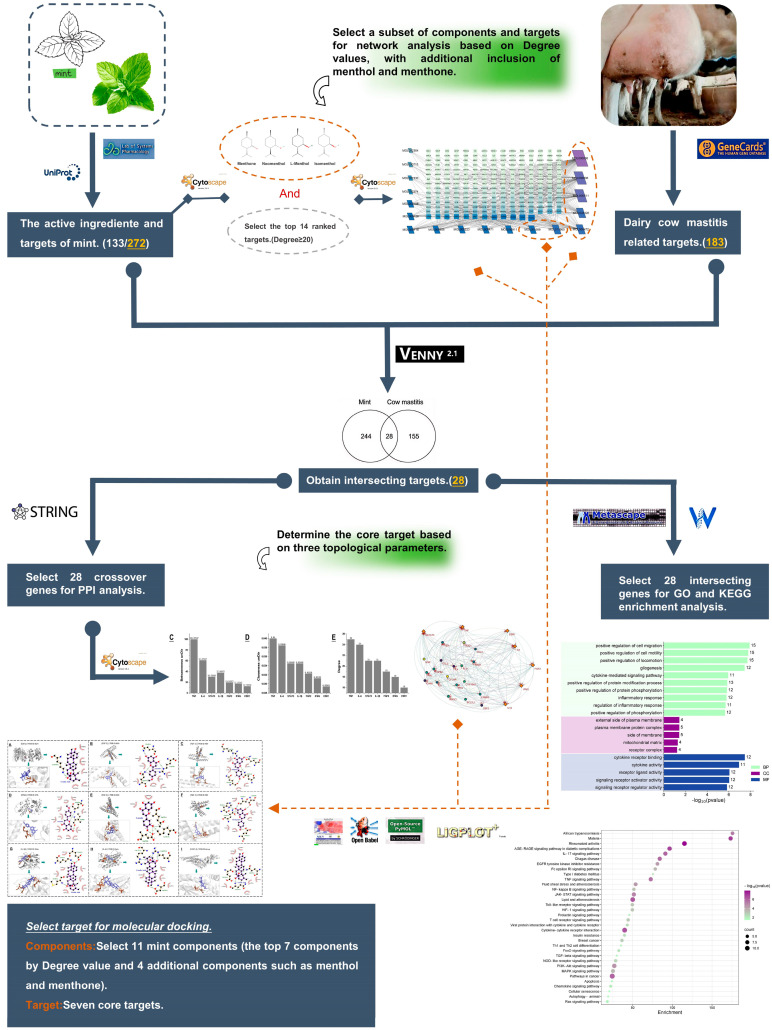
The figure shows the entire content and process of this study, including screening of peppermint components, screening of disease targets, determination of core targets, GO and KEGG enrichment analysis, and molecular docking.

The Traditional Chinese Medicine Systems Pharmacology (TCMSP) database (https://www.tcmsp-e.com/#/database (accessed on 3 December 2024)) was employed to identify the chemical constituents of peppermint and the associated target names for each constituent, which were then imported into Excel to eliminate duplicates. Subsequently, the target names were mapped to their official gene names using the UniProt database (https://www.uniprot.org/ (accessed on 3 December 2024)). Notably, due to database limitations, the species was not exclusively restricted to “Bovine”, with some gene sources being of “Human” origin. The peppermint components and their corresponding targets were visualized using Cytoscape 3.10.0 software (San Diego, CA, USA) to construct a network graph, which aids in the clear depiction of the key components and targets of peppermint. The specific research process is outlined in the accompanying flow chart (Figure 1).

### 2.2. Screening of Potential Targets for Mint Treatment of Bovine Mastitis

Bovine mastitis–related targets were searched using the GeneCards database (https://www.genecards.org/ (accessed on 5 December 2024)), and the results were imported into Excel to remove duplicates. Subsequently, the Venny 2.1 online tool (https://bioinfogp.cnb.csic.es/tools/venny/ (accessed on 5 December 2024)) was utilized to process the targets of peppermint and those related to bovine mastitis, identifying the intersecting targets (Mint–Cow mastitis targets). These targets were considered as potential targets for the treatment of bovine mastitis with peppermint.

### 2.3. Construction of Mint–Cow Mastitis Targets Protein–Protein Interaction Network

To further investigate the interactions between peppermint- and bovine mastitis-related targets, the UniProt IDs of the “Mint–Cow mastitis targets” were imported into the STRING database (https://cn.string-db.org/ (accessed on 8 December 2024)) to generate a protein–protein interaction (PPI) network. The TSV file was then imported into Cytoscape 3.10.0 software to construct the PPI network model. Utilizing three topological parameters within the network—Betweenness unDir, Closeness unDir, and Degree unDir—the core targets were identified by selecting genes that met or exceeded the thresholds for these parameters. The remaining targets were ranked based on their Degree values. To examine the network connections between core targets and peppermint components, the editable file of the mint component-target network graph, which was created in “Section 2.1”, was reopened using Cytoscape 3.10.0 software. The core targets and their associated peppermint components were extracted and assigned to a new group for individual analysis and visualization.

### 2.4. Gene Ontology (GO) Enrichment and KEGG Pathway Enrichment Analysis

The “Mint–Cow mastitis targets” were imported into the Metascape database (https://metascape.org/gp/index.html#/main/step1 (accessed on 9 December 2024)) for enrichment analysis, utilizing all genes as the background for enrichment. Datasets for KEGG pathways, GO biological processes (BPs), GO cellular components (CCs), and GO molecular functions (MFs) were obtained. All entries were sorted in descending order according to their *p*-value and visualized using the “SRplot” Online Platform (https://www.bioinformatics.com.cn/srplot (accessed on 9 December 2024)).

### 2.5. Molecular Docking Analysis

According to the screening results of network pharmacology mentioned earlier, the 3D structures of target proteins were obtained from the PDB database (https://www.rcsb.org/ (accessed on 10 December 2024)) and the 3D structures of key peppermint small–molecule compounds were obtained from the PubChem database (https://pubchem.ncbi.nlm.nih.gov/ (accessed on 10 December 2024)). Each screened key small–molecule compound was required to undergo molecular docking with different key targets. The target proteins and small–molecule compounds were processed using PyMOL 2.6.0a0, which included the removal of water molecules and the addition of hydrogen atoms. Subsequently, the processed PDB–format files were imported into AutoDockTools–1.5.6 (La Jolla, CA, USA), with the proteins being designated as receptors. The docking box was designed to encompass the entire protein structure, thereby ensuring that no potential binding sites were overlooked. To assess the docking activity of the targets with peppermint small molecules, molecular docking simulations were conducted using a semi–flexible docking mode in AutoDock 4. Each docking outputs 10 results. The docking binding energy should be primarily evaluated. When the binding energies are similar, priority should be given to a conformation with a higher number of hydrogen bonds. According to the above criteria, the best docking result was selected from the 10 results and saved in PDBQT format. Subsequently, OpenBabel–2.4.0–x86 was used to convert the file to PDB format for visualization using PyMOL 2.6.0a0 and LigPlus 2.2.8.

## 3. Results

### 3.1. The Active Ingredients of Peppermint and Their Corresponding Targets

**Figure 2:**
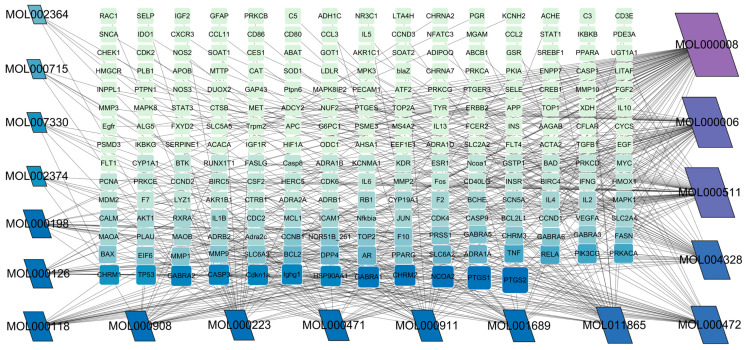
Mint ingredient–target point network diagram. Trapezoids represent the components of mint, while rectangles denote the corresponding targets for each component. Mint components are indicated by MOL IDs, and each edge signifies an interaction, with color gradients from dark to light representing the degree values.

Using the TCMSP database, the chemical components of peppermint were retrieved without applying any filtering criteria, resulting in a total of 164 active components and 1392 associated target points. After removing duplicates, 133 active components and 272 target points were obtained. For the convenience of graphic display, a portion of peppermint components and their corresponding targets were selected for network graph construction. Initially, all ingredients and targets were imported into Cytoscape 3.10.0 software. The degree values were subsequently analyzed and sorted. The top 14 active ingredients with the highest degree rankings were then selected. In addition to these, menthol, menthone, isomenthol, and neomenthol were included, which are reported to be the main components of peppermint oil and possess anti–inflammatory and analgesic effects. Ultimately, 18 active components and 223 targets were chosen for network construction (Figure 2). The network comprises 241 nodes and 502 edges, with 18 nodes originating from components and the remainder from targets. The degree values and molecular structures of some peppermint components were summarized (Table 1).

### 3.2. Acquisition of Potential Targets for Mint Treatment of Bovine Mastitis and Construction of PPI Network

**Figure 3:**
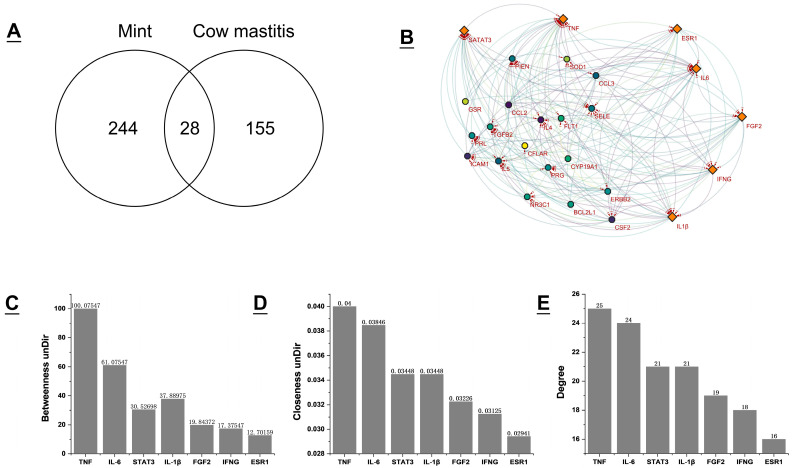
Potential target analysis of mint in treating mastitis in dairy cows. (**A**) Venn diagram of potential targets for the mint treatment of bovine mastitis. (**B**) In the visualized interaction network, orange diamonds represent the core targets, while the remaining circles represent other targets. The shade of color indicates the Degree value, with darker colors signifying a higher Degree. (**C**) The Betweenness unDir values of the core target are displayed, all of which are greater than the threshold (12.230769230769234). (**D**) The Closeness unDir values of the core target are displayed, all of which are greater than the threshold (0.027597822814210176). (**E**) The Degree values of the core target are displayed, all of which are greater than the threshold (12.76923076923077).

**Figure 4:**
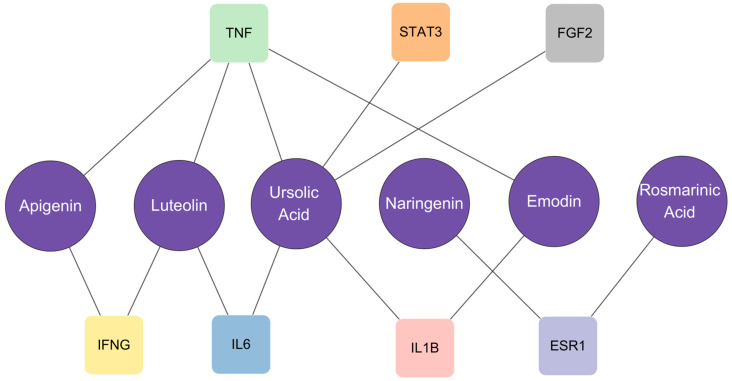
The relationship between 7 core objectives and mint components. Purple circles represent the components of mint, colored squares represent the core targets, and straight lines show the connections between them.

A total of 183 targets related to bovine mastitis were retrieved using the GeneCards database. After comparing these targets with 272 peppermint targets, 28 common targets were obtained (Figure 3A). These targets were imported into the STRING database to analyze and generate a protein–protein interaction (PPI) network, which comprised 28 nodes and 166 edges, with an average node degree of 11.9 and an average local clustering coefficient of 0.752. The PPI network was imported into Cytoscape 3.10.0 software in TSV format to identify core targets, including TNF, IL–6, STAT–3, IL–1β, FGF–2, IFNG, and ESR–1 (Figure 3B). The selection of these core targets primarily relied on three parameters: Betweenness unDir (12.230769230769234), Closeness unDir (0.027597822814210176), and Degree unDir (12.76923076923077), which reflect the nodes’ betweenness centrality, closeness centrality, and degree centrality in the network, respectively. Targets that met or exceeded these thresholds were designated as core targets. Bar charts illustrate the specific values of these core targets under the three parameters (Figure 3C–E). Additionally, a network connection between the aforementioned core targets and critical peppermint components was established (Figure 4). Among the seven core targets, apigenin, luteolin, rosmarinic acid, and emodin are all connected to TNF, indicating a high correlation between TNF and the treatment of bovine mastitis with peppermint. Similarly, ursolic acid is connected to multiple core targets, emphasizing its importance in the treatment process.

### 3.3. GO Function and KEGG Enrichment Analysis

**Figure 5:**
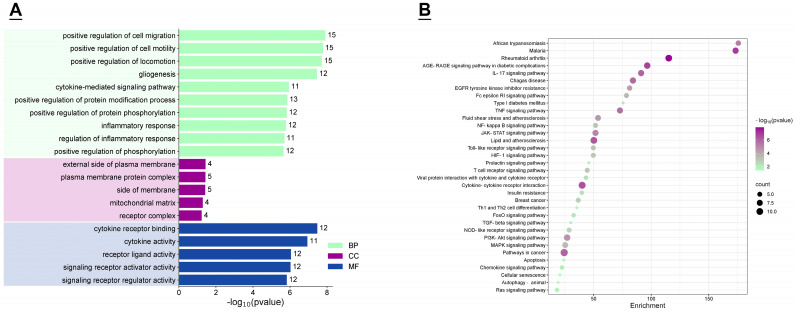
GO and KEGG analysis results. (**A**) The displayed terms are the top ranked biological processes, cellular components, and molecular functions in GO analysis, ranked according to “−log10 (*p*-value).” (**B**) Partial KEGG signaling pathway entries, and the X-axis represents the enrichment score.

Following GO functional analysis, 584 related entries were identified, with 551 belonging to biological processes, 10 to cellular components, and 22 to molecular functions. The results suggest that the Mint–Cow mastitis targets may be involved in biological processes such as positive regulation of cell migration, cytokine–mediated signaling pathway, and inflammatory response. Regarding cellular components, these include the external side of plasma membrane, the mitochondrial matrix, and the receptor complex. These targets also exert molecular functions like cytokine receptor binding, signaling receptor activator activity, and signaling receptor regulator activity (Figure 5A). KEGG analysis enriched a total of 89 pathways, indicating that peppermint may modulate biological responses through pathways such as the AGEs–RAGE signaling pathway, IL–17 signaling pathway, NF–kappa B signaling pathway, Toll–like receptor signaling pathway, HIF–1 signaling pathway, TGF–β signaling pathway, PI3K–Akt signaling pathway, and MAPK signaling pathway (Figure 5B).

### 3.4. Molecular Docking

**Figure 6:**
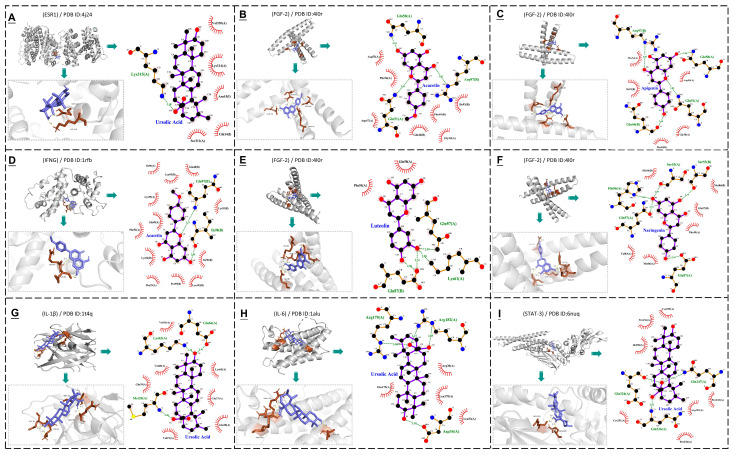
Molecular docking results of peppermint components with core targets. The image in the upper left corner of each region is a macro 3D visualization, the lower left corner shows the details of the 3D visualization, and the 2D visualization is on the right side. (**A**) Docking ursolic acid and ESR–1; (**B**) Docking acacetin and FGF–2; (**C**) Docking apigenin and FGF–2; (**D**) Docking acacetin and IFNG; (**E**) Docking luteolin and FGF–2; (**F**) Docking naringenin and FGF–2; (**G**) Docking ursolic acid and IL–1β; (**H**) Docking ursolic acid and IL–6; (**I**) Docking ursolic acid and STAT–3.

Based on the results of network pharmacology, molecular docking was performed to verify the binding patterns between key compounds of pepermint, such as apigenin, luteolin, ursolic acid, etc., and the seven core targets of bovine mastitis. All docking simulations were performed using a semi–flexible docking approach. Table 2 presents the docking binding energies and the number of hydrogen bonds for the interactions between 11 key compounds of peppermint and seven core targets. Results with docking binding energies less than −6 kcal/mol were selected, and their PDB files were imported into PyMOL 2.6.0a0 and LigPlus 2.2.8 software to generate 3D stereo and 2D planar diagrams, respectively. The diagrams illustrate the binding interactions between the ligands and partial amino acid residues of the receptors, and display the position of hydrogen bonds (Figure 6).

## 4. Discussion

This study utilized network pharmacology and molecular docking techniques to elucidate the potential mechanism of peppermint in treating bovine mastitis. The key findings indicate that the main components of peppermint, such as apigenin, luteolin, and ursolic acid, exhibit good binding affinity with the core targets of bovine mastitis. These results effectively highlight the application value of peppermint in the treatment of mastitis in cows. Mint applications are extensive, serving as a medicinal treatment for gastrointestinal disorders, dermatological conditions, and postoperative adjunct therapy [29,30,31]. As a dietary supplement, it improves functional gastrointestinal diseases [32]. Peppermint is also utilized in the food and cosmetics industries [33,34]. In addition, in the agricultural field, due to its anti–inflammatory and antibacterial properties, it is often added to animal diets for the prevention or treatment of certain diseases [35]. Considering the diverse applications of peppermint, this study searched for its components in the TCMSP database without filtering by the drug–like index (DL), as this would exclude potentially effective active components such as menthol and menthone, which have a DL value of only 0.03. Many studies have investigated the effects of peppermint on anti–inflammatory, antibacterial, antiviral, immune regulation, anti–tumor, neuroprotective, anti–fatigue, and antioxidant activities.

Most research on peppermint as a herbal medicine for mammals emphasizes the biological effects of peppermint volatile extracts (essential oils) [9,36,37]; however, it was found that some active ingredients in peppermint seem to have more connections with the disease targets of bovine mastitis, including flavonoid compounds such as apigenin, luteolin, and naringin; triterpenoid compounds, such as ursolic acid; anthraquinone derivatives, such as emodin; and phenolic acid compounds, such as rosmarinic acid. Apigenin, a natural flavonoid, is known for its antioxidant capabilities and is used as a therapeutic agent to overcome inflammation and autoimmune conditions [38]. Apigenin can participate in the inflammatory response by inhibiting the expression of upstream proteins such as TLR4 and TRAF6, as well as downstream proteins like IL–1β, IL–6, and TNF–α [39]. Pretreatment of SD rat mammary inflammatory cells with apigenin significantly reduces the protein expression of inflammatory factors like IL–1β, IL–6, and TNF–α [40]. Luteolin can inhibit the expression of bacterial soluble proteins and nucleic acid metabolism, exerting an inhibitory effect on Gram–positive bacteria such as *Staphylococcus aureus* and some Gram–negative bacteria [41]. The anti–inflammatory effect of luteolin is also prominent. It can reduce the expression of inflammatory factors such as TNF–α and IL–6 by inhibiting the NF–κB signaling pathway. Additionally, it can decrease the gene transcription and protein expression of MMP–2 and MMP–9 by inhibiting the NF–κB and MAPK pathways, thereby alleviating tissue damage caused by inflammation [42]. Ursolic acid, a common pentacyclic triterpenoid, has been shown to reduce the secretion of inflammatory factors such as IL–1β, IL–6, and TNF–α in LPS–induced macrophage inflammation models, thus playing an anti–inflammatory role [43]. The antimicrobial potentiating effect of ursolic acid is also frequently mentioned; when used in combination with β–lactam antibiotics like ampicillin, it can exhibit synergistic effects, enhancing the antibiotic susceptibility of *Staphylococcus aureus* and *Staphylococcus epidermidis* [44]. A study has successfully alleviated the pathological damage associated with acute pancreatitis (SAP) through the utilization of emodin’s antioxidant and anti–inflammatory capabilities, thereby confirming its advantageous role in anti–inflammatory therapy [45]. Numerous in vivo and in vitro studies have confirmed the anti–inflammatory effects of rosmarinic acid in inflammatory diseases, such as arthritis, colitis, dermatitis, and mastitis [46,47,48,49]. These compounds possess potent anti–inflammatory and antibacterial pharmacological effects, which align with the clinical philosophy for treating bovine mastitis. Therefore, it is inferred that these compounds may play a significant role in the therapeutic action of peppermint against bovine mastitis.

Utilizing the PPI network, the topological parameters of Betweenness unDir, Closeness unDir, and Degree unDir were employed to predict the core targets for mint in treating mastitis in dairy cows, which include TNF, IL–6, STAT–3, IL–1β, FGF–2, IFNG, and ESR–1. Previous studies have indicated that TNF, IL–6, and IL–1β, as pro–inflammatory factors, are highly expressed in cows with mastitis [50,51]. Following LPS treatment of bovine mammary epithelial cells, the mRNA abundance of TNF, IL–6, and IL–1β is also upregulated [52]. The interaction between mint components and core targets (Figure 4) shows that ursolic acid in mint is associated with TNF, IL–6, and IL–1β, reflecting its potential active role in the treatment of mastitis in dairy cows. Although there is scant research on the treatment of mastitis in cows with ursolic acid, its anti–inflammatory effects should not be overlooked. Studies have shown that ursolic acid may prevent sepsis–induced acute kidney injury in mice by inhibiting reactive oxygen species and inflammatory cytokines in the kidneys, including TNF–α, IL–1β, and IL–6 [53]. Ursolic acid can also inhibit the activation of microglia and astrocytes and reduce the levels of TNF–α, IL–1β, and IL–6 in the brain inflammation of mice with lipopolysaccharide–induced cognitive deficits [54]. STAT–3, a member of the signal transducers and activators of transcription family, plays a crucial role in controlling inflammation and participating in immunity in vertebrates [55]. As a downstream pathway of the IL–6/STAT–3 axis, STAT–3 is a hot topic in cancer and inflammation research [56]. It has been found that the absence of the STAT–3 gene in mouse intestinal epithelial cells significantly alleviates the worsening of colitis caused by P2Y13 (G protein–coupled receptor) activation, suggesting that STAT–3 may be a key target for the treatment of ulcerative colitis [57]. It has been proposed that sinomenine hydrochloride may have therapeutic effects on plasma cell mastitis based on its anti–inflammatory and immunomodulatory properties, which are exerted through the downregulation of the IL–6/JAK2/STAT–3 pathway [58]. FGF–2 is reported to be an inflammation–modulating factor. In a mouse asthma model, FGF–2 acts as an inflammation amplifier, promoting the infiltration of inflammatory cells in the mouse airways and recruiting subepithelial neutrophils, leading to excessive secretion of inflammatory mediators [59]. A study found that in lactating cows, the mRNA of FGF is highly expressed in infected mammary quarters compared to healthy ones after intramammary infusion of *Streptococcus uberis*, suggesting that mastitis may induce changes in the transcriptional level of this growth factor [60]. IFNG is the gene name for interferon–γ (IFN–γ), and it has been proven to drive inflammatory mechanisms [61,62,63]. Studies have shown that in naturally occurring subclinical mastitis in cows, the intramammary inflammatory response is driven by IL–1α, IL–4, IL–12, IL–17A, and IFN–γ [64]. Additionally, an increase in IFN–γ reduces arginine levels and activates TLR4–CCL5 signaling, increasing the susceptibility of bovine mammary epithelial cells to *Staphylococcus aureus* [65]. However, IFN–γ has a complex mechanism of action in organisms; therefore, numerous studies have also demonstrated the positive effects of IFN–γ in diseases. For example, it is known that T cells play an important role in protecting the body from pathogen invasion, and the upregulation of important genes such as IFN–γ enhances T cell activity, thereby coordinating the immune response to *Staphylococcus aureus* infection [66]. Other studies have also shown that an increase in IFN–γ enhances the direct killing of *Staphylococcus aureus* by macrophages [67,68,69]. Estrogen receptor α (ESR–1) is a main biomarker and therapeutic target for endocrine therapy in breast cancer, and research on ESR–1 is mostly related to cancer. In the study, all the aforementioned core targets have varying degrees of association with key compounds in mint (Figure 4). Among them, TNF is connected to as many as four core targets, indirectly reflecting the high relevance of TNF to the process of mint in treating mastitis in dairy cows. In addition, IFNG, IL–6, and IL–1β are also associated with at least two mint components, demonstrating that the treatment of mastitis in dairy cows with mint is the result of the synergistic action of multiple components.

GO functional enrichment analysis and KEGG enrichment analysis indicate that the treatment of mastitis in dairy cows with mint involves multiple biological processes and signaling pathways. The AGEs–RAGE pathway may be one of the significant pathways in the treatment of mastitis in dairy cows with mint. Although there is no direct research evidence linking AGEs–RAGE to mastitis in cows, studies have shown that the AGEs–RAGE axis can promote the occurrence of inflammatory responses. The binding of AGEs to RAGE can activate immune cells to produce inflammatory mediators and chemokines, thereby triggering inflammatory reactions [70]. IL–17 is a key inflammatory cytokine that plays a significant role in various disease processes, including inflammation, hypersensitivity reactions, and autoimmune diseases [71]. IL–17 can activate the NF–κB signaling pathway, leading to the transcription of downstream signaling factors and participating in the body’s inflammatory response [72]. Research suggests that IL–17 may play a key role in the development of mastitis in cows and goats [73,74]. NF–κB, a nuclear transcription factor, is ubiquitous in eukaryotic cells and can regulate immune responses, inflammatory reactions, cell differentiation, apoptosis, and tumor growth [75,76]. Increased activity of NF–κB has been observed in both milk somatic cells and mammary epithelial cells from cows with mastitis [77,78]. Numerous studies on mouse, rat, and bovine mammary epithelial cells have shown that many compounds can improve inflammatory responses by inhibiting signaling pathways such as NF–κB [79,80,81,82,83]. Toll–like receptors (TLRs), as upstream receptors of NF–κB, play an essential role in the immune response to various intracellular pathogens [84]. There are 10 to 15 types of TLRs in most mammalian species, and 10 functional TLRs (TLR1 to TLR10) have been identified in cattle [85,86]. Studies have shown that Mycobacterium bovis BCG activates the ERK1/2 MAPK pathway through TLR2 and TLR4, promoting the secretion of CXCL8 and enhancing the host’s inflammatory response [87]. Hypoxia–inducible factor–1α (HIF–1α) is a key transcriptional regulator of immunity and inflammation. Taurine can modulate HIF–1α, reduce inflammation and mammary tissue damage, and prevent mastitis dysfunction induced by *Streptococcus uberis* [88]. Mammary fibrosis in cows is a common pathological process associated with mastitis. The abnormal upregulation of Transforming Growth Factor–β1 (TGF–β1) may play an important role in bovine mammary fibrosis [89]. The PI3K–Akt pathway is the main stem of various cellular pathways and has some cross–linking effects with the MAPK and NF–κB signaling pathways. Studies have shown that the activation of the PI3K/Akt/mTOR pathway can reduce the inflammatory response of *Staphylococcus aureus*–induced bovine mammary epithelial cells [90]. Inhibition of this pathway can reduce the secretion of IL–6, IL–8, and IL–1β, thereby inhibiting the inflammatory response [91]. The MAPK signaling pathway is mainly involved in inflammatory responses, and regulation of this pathway can suppress mammary inflammation [80,81,92]. In summary, the treatment of mastitis in dairy cows with mint may function through AGEs–RAGE, IL–17, NF–κB, TLRs, HIF–1, TGF–β, PI3K–Akt, MAPK, or other signaling pathways. The molecular docking results show that the binding energy of most docking results is less than –5.00 Kcal/mol, and hydrogen bonds are formed. Among them, ursolic acid has good docking activity with seven core targets. Apigenin has good docking activity with STAT–3, IL–1β, FGF–2, IFNG, and ESR–1. Luteolin has good docking activity with IL–1β and FGF–2. Naringenin has good docking activity with FGF–2. Acacetin has good docking activity with FGF–2 and IFNG. Additionally, it was found that FGF–2 forms stable combinations with many mint components, including apigenin, luteolin, ursolic acid, naringenin, and acacetin. These results suggest that key components of mint such as ursolic acid, apigenin, luteolin, naringenin, and acacetin have good anti–inflammatory activity and may be considered as potential candidates for the development of anti–inflammatory drugs. Considering that FGF–2 can form stable complexes with multiple mint components, FGF–2 can be further studied as a potential target for mint in the treatment of mastitis in dairy cows.

In summary, we have elucidated the potential active components, targets, and molecular mechanisms of peppermint in treating bovine mastitis through network pharmacology and molecular docking. The limitations of our study lie in the fact that the entire process is mainly based on computer simulation and database analysis, thus lacking experimental validation. Our future work will involve in vitro and in vivo experiments to verify these predicted results. Moreover, there are other components in peppermint that may have therapeutic effects, and their mechanisms of action and synergistic effects also need further exploration.

## 5. Conclusions

This study employed network pharmacology and molecular docking techniques to predict the key active components of mint in treating mastitis in dairy cows, including apigenin, luteolin, and ursolic acid. The core targets were identified as TNF, IL–6, STAT–3, IL–1β, FGF–2, IFNG, and ESR–1. KEGG enrichment analysis revealed that the key components of mint can participate in the regulation of the AGEs–RAGE signaling pathway, IL–17 signaling pathway, NF–κB signaling pathway, TLRs signaling pathway, HIF–1 signaling pathway, TGF–β signaling pathway, PI3K–Akt signaling pathway, and MAPK signaling pathway. Molecular docking demonstrated that ursolic acid has good docking activity with all core targets. Additionally, other components also formed stable complexes with some core targets. In summary, the main components of mint can bind to proteins corresponding to targets such as TNF, IL–6, STAT–3, IL–1β, FGF–2, IFNG, and ESR–1, and regulate multiple inflammation–related signaling pathways to exert anti–inflammatory effects. Therefore, mint can play a positive role in the treatment of mastitis in dairy cows. This study provides a theoretical basis for the use of mint in treating mastitis in dairy cows and offers insights for the development and utilization of other natural medicines. In the development of mint and its main components, they can be considered as potential new drugs for the treatment of mastitis in dairy cows.

## Figures and Tables

**Table 1:** Information on some active compounds of peppermint.

MOL ID	Active Ingredient	Degree	Molecule Structure
MOL000008	Apigenin	77	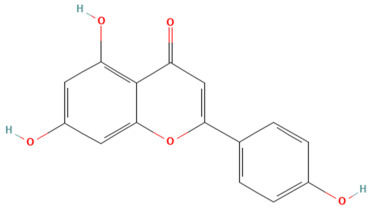
MOL000006	Luteolin	56	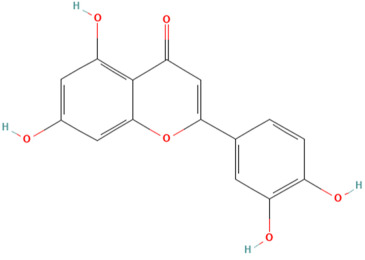
MOL000511	Ursolic Acid	55	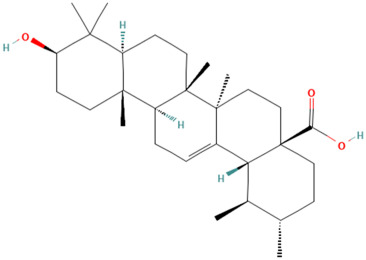
MOL004328	Naringenin	37	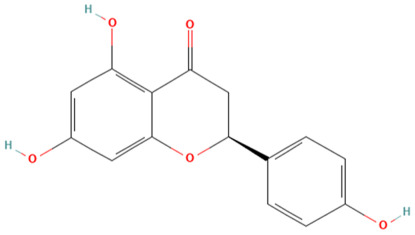
MOL000472	Emodin	35	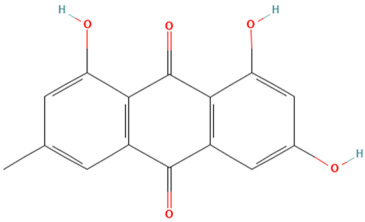
MOL011865	Rosmarinic Acid	32	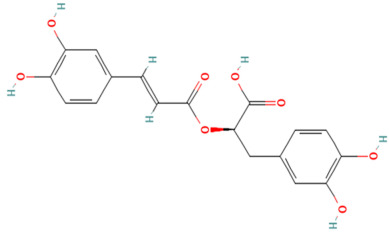
MOL001689	Acacetin	26	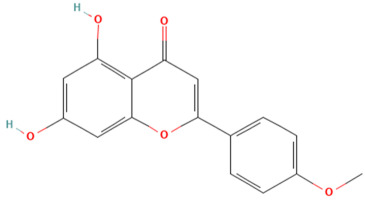
MOL000715	Menthone	12	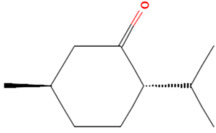
MOL002374	Neomenthol	7	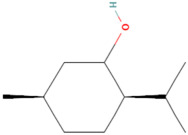
MOL007330	L–Menthol	7	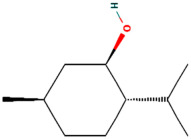
MOL002364	Isomenthol	5	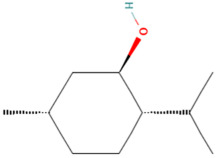

The table includes compounds with higher degree values, as well as Menthone, Neomenthol, L–Menthol and Isomenthol. It presents their degree value information along with their 2D molecular structure information.

**Table 2:** Molecular docking binding energy and hydrogen bond quantity between key components of peppermint and core target.

Mint Components	Core Target (PDB ID)						
	TNF (1u5y)	IL–6 (1alu)	STAT–3 (6nuq)	IL–1β (1t4q)	FGF–2 (4l0r)	IFNG (1rfb)	ESR–1 (4j24)
Apigenin	−4.61/3	−4.3/2	−5.05/2	−5.5/2	−6.71/4	−5.63/2	−5.27/3
Luteolin	−4.27/3	−3.75/1	−4.41/3	−5.3/4	−6.32/5	−4.27/2	−4.16/3
Ursolic Acid	−5.86/2	−6.13/3	−7.45/2	−7.72/2	−5.84/1	−5.78/1	−7.16/1
Naringenin	−5.13/2	−4.09/1	−5.06/2	−5.23/2	−6.51/3	−5.38/1	−5.29/0
Emodin	−4.6/1	−4.81/2	−4.37/1	−5.35/3	−5.53/4	−4.56/1	−4.29/2
Rosmarinic Acid	−2.01/4	−3.12/3	−2.17/1	−2.76/2	−3.12/3	−4.59/2	−2.76/1
Acacetin	−4.64/2	−4.38/1	−4.58/2	−5.35/2	−6.85/3	−6.67/1	−4.38/2
Menthone	−4.72/1	−4.03/1	−4.55/1	−4.22/0	−5.18/1	−5.56/0	−4.06/1
Neomenthol	−4.43/2	−4.19/2	−4.68/2	−4.63/1	−4.97/1	−5.41/2	−5.13/2
L–Menthol	−4.58/1	−3.81/1	−4.33/2	−4.4/1	−5.54/2	−5.35/1	−4.98/1
Isomenthol	−4.39/2	−3.82/2	−4.84/2	−4.23/2	−4.96/1	−5.28/2	−4.79/2

Docking and combining energy (Kcal/mol)/number of hydrogen bonds.

## Data Availability

Data are contained within the article.

## References

[1] ECD/FAO Agricultural Outlook 2022–2031. https://www.fao.org/4/CA4076EN/CA4076EN_Chapter7_Dairy.pdf.

[2] Tricarico J.M., Kebreab E., Wattiaux M.A. (2020). MILK Symposium review: Sustainability of dairy production and consumption in low–income countries with emphasis on productivity and environmental impact. J. Dairy Sci..

[3] Dalanezi F.M., Joaquim S.F., Guimarães F.F., Guerra S.T., Lopes B.C., Schmidt E.M.S., Cerri R.L.A., Langoni H. (2020). Influence of pathogens causing clinical mastitis on reproductive variables of dairy cows. J. Dairy Sci..

[4] Ozbey G., Cambay Z., Yilmaz S., Aytekin O., Zigo F., Ozçelik M., Otlu B. (2022). Identification of bacterial species in milk by MALDI–TOF and assessment of some oxidant–antioxidant parameters in blood and milk from cows with different health status of the udder. Pol. J. Vet. Sci..

[5] Dyson R., Charman N., Hodge A., Rowe S.M., Taylor L.F. (2022). A survey of mastitis pathogens including antimicrobial susceptibility in southeastern Australian dairy herds. J. Dairy Sci..

[6] Rollin E., Dhuyvetter K.C., Overton M.W. (2015). The cost of clinical mastitis in the first 30 days of lactation: An economic modeling tool. Prev. Vet. Med..

[7] Zigo F., Farkašová Z., Výrostková J., Regecová I., Ondrašovičová S., Vargová M., Sasáková N., Pecka–Kielb E., Bursová Š., Kiss D.S. (2022). Dairy Cows’ Udder Pathogens and Occurrence of Virulence Factors in Staphylococci. Animals.

[8] Lopes T.S., Fontoura P.S., Oliveira A., Rizzo F.A., Silveira S., Streck A.F. (2020). Use of plant extracts and essential oils in the control of bovine mastitis. Res. Vet. Sci..

[9] Arbab S., Ullah H., Bano I., Li K., Ul Hassan I., Wang W., Qadeer A., Zhang J. (2022). Evaluation of in vitro antibacterial effect of essential oil and some herbal plant extract used against mastitis pathogens. Vet. Med. Sci..

[10] Zhao H., Ren S., Yang H., Tang S., Guo C., Liu M., Tao Q., Ming T., Xu H. (2022). Peppermint essential oil: Its phytochemistry, biological activity, pharmacological effect and application. Biomed. Pharmacother..

[11] Shanmuganathan R., Hoang Le Q., Devanesan S., Sayed S.R.M., Rajeswari V.D., Liu X., Jhanani G.K. (2023). Mint leaves (*Mentha arvensis*) mediated CaO nanoparticles in dye degradation and their role in anti–inflammatory, anti–cancer properties. Environ. Res..

[12] Azad A.K., Doolaanea A.A., Al-Mahmood S.M.A., Kennedy J.F., Chatterjee B., Bera H. (2021). Electro–hydrodynamic assisted synthesis of lecithin–stabilized peppermint oil–loaded alginate microbeads for intestinal drug delivery. Int. J. Biol. Macromol..

[13] Valková V., Ďúranová H., Galovičová L., Vukovic N.L., Vukic M., Kačániová M. (2021). In Vitro Antimicrobial Activity of Lavender, Mint, and Rosemary Essential Oils and the Effect of Their Vapours on Growth of *Penicillium* spp. in a Bread Model System. Molecules.

[14] De Sousa A.A., Soares P.M., de Almeida A.N., Maia A.R., de Souza E.P., Assreuy A.M. (2010). Antispasmodic effect of *Mentha piperita* essential oil on tracheal smooth muscle of rats. J. Ethnopharmacol..

[15] Shahbazi Y. (2015). Chemical Composition and In Vitro Antibacterial Activity of *Mentha spicata* Essential Oil against Common Food–Borne Pathogenic Bacteria. J. Pathog..

[16] Mimica-Dukic N., Bozin B. (2008). *Mentha* L. species (Lamiaceae) as promising sources of bioactive secondary metabolites. Curr. Pharm. Des..

[17] Ranjbar M., Kiani M., Nikpay A. (2020). Antioxidant and scolicidal activities of four Iranian *Mentha* species (Lamiaceae) in relation to phenolic element. J. Herbmed. Pharmacol..

[18] Li Y., Liu Y., Ma A., Bao Y., Wang M., Sun Z. (2017). In vitro antiviral, anti–inflammatory, and antioxidant activities of the ethanol extract of *Mentha piperita* L.. Food Sci. Biotechnol..

[19] Lang M., Ferron P.J., Bursztyka J., Montjarret A., Duteil E., Bazire A., Bedoux G. (2019). Evaluation of immunomodulatory activities of essential oils by high content analysis. J. Biotechnol..

[20] Harnly J.M., Doherty R.F., Beecher G.R., Holden J.M., Haytowitz D.B., Bhagwat S., Gebhardt S. (2006). Flavonoid content of, U.S. fruits, vegetables, and nuts. J. Agric. Food Chem..

[21] Tutunchi H., Naeini F., Ostadrahimi A., Hosseinzadeh-Attar M.J. (2020). Naringenin, a flavanone with antiviral and anti–inflammatory effects: A promising treatment strategy against COVID–19. Phytother. Res..

[22] Sun L.C., Zhang H.B., Gu C.D., Guo S.D., Li G., Lian R., Yao Y., Zhang G.Q. (2018). Protective effect of acacetin on sepsis–induced acute lung injury via its anti–inflammatory and antioxidative activity. Arch. Pharm. Res..

[23] Al-Kuraishy H.M., Al-Gareeb A.I., Negm W.A., Alexiou A., Batiha G.E. (2022). Ursolic acid and SARS-CoV-2 infection: A new horizon and perspective. Inflammopharmacology.

[24] Luan M., Wang H., Wang J., Zhang X., Zhao F., Liu Z., Meng Q. (2022). Advances in anti–inflammatory Activity, Mechanism and Therapeutic Application of Ursolic Acid. Mini Rev. Med. Chem..

[25] Pollier J., Goossens A. (2012). Oleanolic acid. Phytochemistry.

[26] Huerta-Madroñal M., Caro-León J., Espinosa-Cano E., Aguilar M.R., Vázquez-Lasa B. (2021). Chitosan—Rosmarinic acid conjugates with antioxidant, anti–inflammatory and photoprotective properties. Carbohydr. Polym..

[27] Paciello F., Di Pino A., Rolesi R., Troiani D., Paludetti G., Grassi C., Fetoni A.R. (2020). anti–oxidant and anti–inflammatory effects of caffeic acid: In vivo evidences in a model of noise–induced hearing loss. Food Chem. Toxicol..

[28] Li S., Zhang B. (2013). Traditional Chinese medicine network pharmacology: Theory, methodology and application. Chin. J. Nat. Med..

[29] Weerts Z.Z.R.M., Keszthelyi D., Vork L., Aendekerk N.C.P., Frijlink H.W., Brouwers J.R.B.J., Neef C., Jonkers D.M.A.E., Masclee A.A.M. (2018). A Novel Ileocolonic Release Peppermint Oil Capsule for Treatment of Irritable Bowel Syndrome: A Phase I Study in Healthy Volunteers. Adv. Ther..

[30] Akhavan Amjadi M., Mojab F., Kamranpour S.B. (2012). The effect of peppermint oil on symptomatic treatment of pruritus in pregnant women. Iran. J. Pharm. Res..

[31] Karsten M., Prince D., Robinson R., Stout–Aguilar J. (2020). Effects of Peppermint Aromatherapy on Postoperative Nausea and Vomiting. J. PeriAnesth. Nurs..

[32] Van Tilburg M.A., Felix C.T. (2013). Diet and functional abdominal pain in children and adolescents. J. Pediatr. Gastroenterol. Nutr..

[33] Ibrahim O.A.E., Mohamed A.G., Bahgaat W.K. (2019). Natural peppermint–flavored cheese. Acta Sci. Pol. Technol. Aliment..

[34] Kaur C.D., Saraf S. (2010). In vitro sun protection factor determination of herbal oils used in cosmetics. Pharmacogn. Res..

[35] Hejna M., Kovanda L., Rossi L., Liu Y. (2021). Mint Oils: In Vitro Ability to Perform anti–Inflammatory, Antioxidant, and Antimicrobial Activities and to Enhance Intestinal Barrier Integrity. Antioxidants.

[36] Castillo-Lopez E., Rivera-Chacon R., Ricci S., Petri R.M., Reisinger N., Zebeli Q. (2021). Short–term screening of multiple phytogenic compounds for their potential to modulate chewing behavior, ruminal fermentation profile, and pH in cattle fed grain–rich diets. J. Dairy Sci..

[37] Saha S., Lachance S. (2019). Effect of essential oils on cattle gastrointestinal nematodes assessed by egg hatch, larval migration and mortality testing. J. Helminthol..

[38] Ali F., Rahul N.F., Jyoti S., Siddique Y.H. (2016). Health functionality of apigenin: A review. Int. J. Food Prop..

[39] Vinh L.B., Jang H.J., Phong N.V., Cho K., Park S.S., Kang J.S., Kim Y.H., Yang S.Y. (2019). Isolation, structural elucidation, and insights into the anti–inflammatory effects of triterpene saponins from the leaves of *Stauntonia hexaphylla*. Bioorg. Med. Chem. Lett..

[40] Mantawy E.M., Said R.S., Abdel-Aziz A.K. (2019). Mechanistic approach of the inhibitory effect of chrysin on inflammatory and apoptotic events implicated in radiation–induced premature ovarian failure: Emphasis on TGF–β/MAPKs signaling pathway. Biomed. Pharmacother..

[41] Qiu J., Li H., Meng H., Hu C., Li J., Luo M., Dong J., Wang X., Wang J., Deng Y. (2011). Impact of luteolin on the production of alpha–toxin by Staphylococcus aureus. Lett. Appl. Microbiol..

[42] Guo Y.F., Xu N.N., Sun W., Zhao Y., Li C.Y., Guo M.Y. (2017). Luteolin reduces inflammation in Staphylococcus aureus–induced mastitis by inhibiting NF–kB activation and MMPs expression. Oncotarget.

[43] Li J., Li N., Yan S., Liu M., Sun B., Lu Y., Shao Y. (2018). Ursolic acid alleviates inflammation and against diabetes–induced nephropathy through TLR4–mediated inflammatory pathway. Mol. Med. Rep..

[44] Kurek A., Nadkowska P., Pliszka S., Wolska K.I. (2012). Modulation of antibiotic resistance in bacterial pathogens by oleanolic acid and ursolic acid. Phytomedicine.

[45] Xia S., Ni Y., Zhou Q., Liu H., Xiang H., Sui H., Shang D. (2019). Emodin Attenuates Severe Acute Pancreatitis via Antioxidant and anti–inflammatory Activity. Inflammation.

[46] Hu Z.N., Huang L.J., Chen W.P. (2018). The inhibitory effects of rosmarinic acid on catabolism induced by IL–1β in rat chondrocyte. Acta Biochim. Pol..

[47] Zhao L., Zhang Y., Liu G., Hao S., Wang C., Wang Y. (2018). Black rice anthocyanin–rich extract and rosmarinic acid, alone and in combination, protect against DSS–induced colitis in mice. Food Funct..

[48] Lee J., Jung E., Koh J., Kim Y.S., Park D. (2008). Effect of rosmarinic acid on atopic dermatitis. J. Dermatol..

[49] Jiang K., Ma X., Guo S., Zhang T., Zhao G., Wu H., Wang X., Deng G. (2018). anti–inflammatory Effects of Rosmarinic Acid in Lipopolysaccharide–Induced Mastitis in Mice. Inflammation.

[50] Akhtar M., Guo S., Guo Y.F., Zahoor A., Shaukat A., Chen Y., Umar T., Deng P.G., Guo M. (2020). Upregulated–gene expression of pro–inflammatory cytokines (TNF–α, IL–1β and IL–6) via TLRs following NF–κB and MAPKs in bovine mastitis. Acta Trop..

[51] Johnzon C.F., Dahlberg J., Gustafson A.M., Waern I., Moazzami A.A., Östensson K., Pejler G. (2018). The Effect of Lipopolysaccharide–Induced Experimental Bovine Mastitis on Clinical Parameters, Inflammatory Markers, and the Metabolome: A Kinetic Approach. Front. Immunol..

[52] Liu S., Guo W., Jia Y., Ye B., Liu S., Fu S., Liu J., Hu G. (2021). Menthol Targeting AMPK Alleviates the Inflammatory Response of Bovine Mammary Epithelial Cells and Restores the Synthesis of Milk Fat and Milk Protein. Front. Immunol..

[53] Zhang Z., Zhang H., Chen R., Wang Z. (2018). Oral supplementation with ursolic acid ameliorates sepsis–induced acute kidney injury in a mouse model by inhibiting oxidative stress and inflammatory responses. Mol. Med. Rep..

[54] Wang Y.J., Lu J., Wu D.M., Zheng Z.H., Zheng Y.L., Wang X.H., Ruan J., Sun X., Shan Q., Zhang Z.F. (2011). Ursolic acid attenuates lipopolysaccharide–induced cognitive deficits in mouse brain through suppressing p38/NF–κB mediated inflammatory pathways. Neurobiol. Learn. Mem..

[55] Hillmer E.J., Zhang H., Li H.S., Watowich S.S. (2016). STAT3 signaling in immunity. Cytokine Growth Factor. Rev..

[56] Song Z., Ren D., Xu X., Wang Y. (2018). Molecular cross–talk of IL–6 in tumors and new progress in combined therapy. Thorac. Cancer.

[57] Wu X., Wei S., Chen M., Li J., Wei Y., Zhang J., Dong W. (2022). P2RY13 Exacerbates Intestinal Inflammation by Damaging the Intestinal Mucosal Barrier via Activating IL–6/STAT3 Pathway. Int. J. Biol. Sci..

[58] Liu Y., Sun Y., Zhou Y., Tang X., Wang K., Ren Y., He J. (2020). Sinomenine hydrochloride inhibits the progression of plasma cell mastitis by regulating IL–6/JAK2/STAT3 pathway. Int. Immunopharmacol..

[59] Tan Y.Y., Zhou H.Q., Lin Y.J., Yi L.T., Chen Z.G., Cao Q.D., Guo Y.R., Wang Z.N., Chen S.D., Li Y. (2022). FGF2 is overexpressed in asthma and promotes airway inflammation through the FGFR/MAPK/NF–κB pathway in airway epithelial cells. Mil. Med. Res..

[60] Sheffield L.G. (1997). Mastitis increases growth factor messenger ribonucleic acid in bovine mammary glands. J. Dairy Sci..

[61] Lee E., Chanamara S., Pleasure D., Soulika A.M. (2012). IFN–gamma signaling in the central nervous system controls the course of experimental autoimmune encephalomyelitis independently of the localization and composition of inflammatory foci. J. Neuroinflamm..

[62] Legroux L., Arbour N. (2015). Multiple Sclerosis and T Lymphocytes: An Entangled Story. J. Neuroimmune Pharmacol..

[63] Kuchroo V.K., Anderson A.C., Waldner H., Munder M., Bettelli E., Nicholson L.B. (2002). T cell response in experimental autoimmune encephalomyelitis (EAE): Role of self and cross–reactive antigens in shaping, tuning, and regulating the autopathogenic T cell repertoire. Annu. Rev. Immunol..

[64] Vitenberga-Verza Z., Pilmane M., Šerstņova K., Melderis I., Gontar Ł., Kochański M., Drutowska A., Maróti G., Prieto–Simón B. (2022). Identification of Inflammatory and Regulatory Cytokines IL–1α–, IL–4–, IL–6–, IL–12–, IL–13–, IL–17A–, TNF–α–, and IFN–γ–Producing Cells in the Milk of Dairy Cows with Subclinical and Clinical Mastitis. Pathogens.

[65] Liu B., Che Y., Zhang M., Ren W., Xia X., Liu H., Huang T., Huang J., Lei L. (2020). IFN–γ Activates the TLR4–CCL5 Signaling Through Reducing Arginine Level, Leading to Enhanced Susceptibility of Bovine Mammary Epithelial Cells to Staphylococcus aureus. Inflammation.

[66] Wang M., Bissonnette N., Laterrière M., Dudemaine P.L., Gagné D., Roy J.P., Sirard M.A., Ibeagha–Awemu E.M. (2023). Gene co–expression in response to Staphylococcus aureus infection reveals networks of genes with specific functions during bovine subclinical mastitis. J. Dairy Sci..

[67] Zhao Y.X., Nilsson I.M., Tarkowski A. (1998). The dual role of interferon–gamma in experimental Staphylococcus aureus septicaemia versus arthritis. Immunology.

[68] Nguyen Q.T., Furuya Y., Roberts S., Metzger D.W. (2015). Role of Interleukin–12 in Protection against Pulmonary Infection with Methicillin–Resistant Staphylococcus aureus. Antimicrob. Agents Chemother..

[69] Greenlee-Wacker M.C., Nauseef W.M. (2017). IFN–γ targets macrophage–mediated immune responses toward Staphylococcus aureus. J. Leukoc. Biol..

[70] Zhou M., Zhang Y., Shi L., Li L., Zhang D., Gong Z., Wu Q. (2024). Activation and modulation of the AGEs–RAGE axis: Implications for inflammatory pathologies and therapeutic interventions—A review. Pharmacol. Res..

[71] Rubino S.J., Geddes K., Girardin S.E. (2012). Innate IL–17 and IL–22 responses to enteric bacterial pathogens. Trends Immunol..

[72] Bechara R., McGeachy M.J., Gaffen S.L. (2021). The metabolism–modulating activity of IL–17 signaling in health and disease. J. Exp. Med..

[73] Tao W., Mallard B. (2007). Differentially expressed genes associated with Staphylococcus aureus mastitis of Canadian Holstein cows. Vet. Immunol. Immunopathol..

[74] Pisoni G., Moroni P., Genini S., Stella A., Boettcher P.J., Cremonesi P., Scaccabarozzi L., Giuffra E., Castiglioni B. (2010). Differentially expressed genes associated with Staphylococcus aureus mastitis in dairy goats. Vet. Immunol. Immunopathol..

[75] Bidère N., Ngo V.N., Lee J., Collins C., Zheng L., Wan F., Davis R.E., Lenz G., Anderson D.E., Arnoult D. (2009). Casein kinase 1alpha governs antigen–receptor–induced NF–kappaB activation and human lymphoma cell survival. Nature.

[76] Vallabhapurapu S., Karin M. (2009). Regulation and function of NF–kappaB transcription factors in the immune system. Annu. Rev. Immunol..

[77] Boulanger D., Bureau F., Mélotte D., Mainil J., Lekeux P. (2003). Increased nuclear factor kappaB activity in milk cells of mastitis–affected cows. J. Dairy Sci..

[78] Boutet P., Sulon J., Closset R., Detilleux J., Beckers J.F., Bureau F., Lekeux P. (2007). Prolactin–induced activation of nuclear factor kappaB in bovine mammary epithelial cells: Role in chronic mastitis. J. Dairy Sci..

[79] Bao L., Sun H., Zhao Y., Feng L., Wu K., Shang S., Xu J., Shan R., Duan S., Qiu M. (2023). Hexadecanamide alleviates Staphylococcus aureus–induced mastitis in mice by inhibiting inflammatory responses and restoring blood–milk barrier integrity. PLoS Pathog..

[80] Zhou G., Zhang W., Wen H., Su Q., Hao Z., Liu J., Gao Y., Zhang H., Ge B., Tong C. (2023). Esculetin improves murine mastitis induced by streptococcus isolated from bovine mammary glands by inhibiting NF–κB and MAPK signaling pathways. Microb. Pathog..

[81] Zhang D., Jin G., Liu W., Dou M., Wang X., Shi W., Bao Y. (2022). Salvia miltiorrhiza polysaccharides ameliorates Staphylococcus aureus–induced mastitis in rats by inhibiting activation of the NF–κB and MAPK signaling pathways. BMC Vet. Res..

[82] Meng M., Huo R., Ma N., Chang G., Shen X. (2022). β–carotene alleviates LPS–induced inflammation through regulating STIM1/ORAI1 expression in bovine mammary epithelial cells. Int. Immunopharmacol..

[83] Li C., Li L., Chen K., Wang Y., Yang F., Wang G. (2019). UFL1 Alleviates Lipopolysaccharide–Induced Cell Damage and Inflammation via Regulation of the TLR4/NF–*κ*B Pathway in Bovine Mammary Epithelial Cells. Oxidative Med. Cell Longev..

[84] Jiang K., Chen X., Zhao G., Wu H., Mi J., Qiu C., Peng X., Deng G. (2017). IFN–τ Plays an anti–Inflammatory Role in Staphylococcus aureus–Induced Endometritis in Mice Through the Suppression of NF–κB Pathway and MMP9 Expression. J. Interferon Cytokine Res..

[85] Yang J., Liu Y., Lin C., Yan R., Li Z., Chen Q., Zhang H., Xu H., Chen X., Chen Y. (2022). Regularity of Toll–Like Receptors in Bovine Mammary Epithelial Cells Induced by *Mycoplasma bovis*. Front. Vet. Sci..

[86] Maurić Maljković M., Vlahek I., Piplica A., Ekert Kabalin A., Sušić V., Stevanović V. (2023). Prospects of toll–like receptors in dairy cattle breeding. Anim. Genet..

[87] Méndez-Samperio P., Belmont L., Miranda E. (2008). Mycobacterium bovis BCG Toll–like receptors 2 and 4 cooperation increases the innate epithelial immune response. Arch. Med. Res..

[88] Lan R., Zhou Y., Wang Z., Fu S., Gao Y., Gao X., Zhang J., Han X., Phouthapane V., Xu Y. (2022). Reduction of ROS–HIF1α–driven glycolysis by taurine alleviates *Streptococcus uberis* infection. Food Funct..

[89] Chen Q., Yang W., Wang X., Li X., Qi S., Zhang Y., Gao M.Q. (2017). TGF–β1 Induces EMT in Bovine Mammary Epithelial Cells Through the TGFβ1/Smad Signaling Pathway. Cell Physiol. Biochem..

[90] Geng N., Liu K., Lu J., Xu Y., Wang X., Wang R., Liu J., Liu Y., Han B. (2020). Autophagy of bovine mammary epithelial cell induced by intracellular Staphylococcus aureus. J. Microbiol..

[91] Manosalva C., Quiroga J., Teuber S., Cárdenas S., Carretta M.D., Morán G.G., Alarcón P., Hidalgo M.A., Burgos R.A. (2020). D–Lactate Increases Cytokine Production in Bovine Fibroblast–Like Synoviocytes via MCT1 Uptake and the MAPK, PI3K/Akt, and NFκB Pathways. Animals.

[92] Zhang X., Jia F., Ma W., Li X., Zhou X. (2022). DAD3 targets ACE2 to inhibit the MAPK and NF–κB signalling pathways and protect against LPS–induced inflammation in bovine mammary epithelial cells. Vet. Res..

